# Protective effects of Re-yan-ning mixture on *Streptococcus pneumonia* in rats based on network pharmacology

**DOI:** 10.1080/13880209.2021.1872653

**Published:** 2021-03-07

**Authors:** Lizhu Han, Jing Kou, Kunxia Hu, Yunlan Wang, Zhishu Tang, Zhisheng Wu, Xiao Song

**Affiliations:** a College of Pharmacy, Shaanxi University of Chinese Medicine, Xianyang, China; b College of Pharmacy, Beijing University of Chinese Medicine, Beijing, China

**Keywords:** Traditional Chinese medicine, pneumonia, signal pathway

## Abstract

**Context:**

Re-yan-ning mixture (RYNM) is a new national drug approved by China's State Food and Drug Administration for the treatment of colds, simple pneumonia and acute bronchitis.

**Objective:**

To determine the mechanism of action of RYNM in the treatment of bacterial pneumonia.

**Materials and methods:**

Using the network pharmacology approach, the multiple components, component candidate targets and multiple therapeutic targets of RYNM were screened and functionally enriched. Also, we established a rat *Streptococcus pneumonia* model to verify the results of network pharmacology enrichment analysis. Forty male SPF Sprague Dawley rats were divided into four groups of 10 rats: control (normal saline), model (normal saline), levofloxacin-intervened and RYNM-intervened groups. IL-10, NOS2, COX-1, IL-6, TNF-α and NF-κB in serum and BALF were detected by ELISA. Western blot detected IL-17, IL-6, TNF-α, COX-2 and Bcl-2.

**Results:**

The network pharmacology approach successfully identified 48 bioactive components in RYNM, and 65 potential targets and 138 signal pathways involved in the treatment of *Streptococcus pneumonia* with RYNM. The *in vivo* experiments indicated that model group has visible inflammation and lesions while RYNM and levofloxacin groups have not. The RYNM exhibited its therapeutic effects on *Streptococcus pneumonia* mainly via the regulation of cell proliferation and survival through the IL-6/IL-10/IL-17, Bax/Bcl-2, COX-1/COX-2, NF-κB and TNF-α signalling pathways.

**Discussion and conclusions:**

The present study demonstrated the protective effects of RYNM on *Streptococcus pneumonia*, providing a potential mechanism for the treatment of bacterial pneumonia with RYNM.

## Introduction

Pneumonia refers to inflammation of the terminal airway, alveolar and interstitial lung, and can be caused by pathogenic microorganisms such as bacteria, viruses, fungi, atypical pathogens and the inhalation of foreign bodies (Ho and Ip 2019; McLaughlin et al. 2019). Bacterial pneumonia is the most common form of pneumonia and one of the most common infectious diseases (Voiriot et al. 2019). Before the use of antibiotics, bacterial pneumonia posed a great threat to the health of children and the elderly. However, in recent years, despite the use of powerful antibiotics and effective vaccines, the overall mortality rate of pneumonia has not changed. *Streptococcus pneumonia* is a Gram positive bacterium, usually located in the nasopharyngeal cavity of healthy people. It may cause disease when immunity declines, especially in children <5 years old and in those ≥60 years (Poole and Clark 2020). *Streptococcus pneumonia* is the main cause of bacterial pneumonia and meningitis worldwide, which can lead to bacteraemia, acute otitis media, bronchitis and other diseases. Pneumococcal infection can be treated with penicillin, cephalosporin, etc. Chinese *Streptococcus pneumonia* is highly resistant to macrolide antibacterial drugs. Clinical data from Wuhan Hospital in China showed that the resistance rate of *Streptococcus pneumonia* to clindamycin was 98.28%, and the resistance rate to erythromycin was 97.41% (Jing et al. 2019). It has high sensitivity to ertapenem, levofloxacin, moxifloxacin, ofloxacin, telithromycin and cefotaxime (Xuan et al. 2019).

The Re-yan-ning mixture (RYNM) is a state-level new drug approved by the State Food and Drug Administration, which consists of Dandelion (the whole herb of *Taraxacum mongolicum* Hand.-Mazz [Compositae]), Polygoni Cuspidati Rhizoma (the root and rhizome of *Polygonum cuspidatum* Sieb.et Zucc. [Polygonaceae]), Sonhi Arvensis (the aerial part of *Sonchus oleraceus* (L.) L. [Compositae]) and Scutellariae Barbatae (the aerial part of *Scutellaria barbata* D. Don [Lamiaceae]) in a 2:2:2:1 proportion by weight. It can be used to treat fever, sore throat, simple pneumonia, suppurative tonsillitis, acute pharyngitis, acute bronchitis and other diseases (Wang et al. 2019). RYNM is a pure Chinese medicinal preparation with multiple targets and multi-path synergistic therapeutic effects. It has less toxicity and side effects, can effectively enhance human immunity and has antibacterial, anti-inflammatory and antiviral effects. Traditional Chinese Medicine (TCM) has a complex pharmacological action with multiple components and targets, which is the unique difference between TCM and western medicine (Pei et al. 2013). Network pharmacology emphasizes the integration of biological networks and drug action networks, clarifies the basic theories of TCM research from the perspective of multi-component, multi-target and multi-channel, and provides a new way of thinking in relation to the modernization of TCM (Zhang and Li 2015; Zhao and He 2018). In this study, based on the multi-component, multi-target and multi-channel of TCM, the Chinese medicine system pharmacology database, DisGeNET, DrugBank and KEGG database were searched to induce pneumonia by tracheal instillation of pneumococcal bacteria in rats. Physiological and biochemical indicators and the mechanism of the interaction of active ingredients in the treatment of pneumonia and metabolic pathways were examined to provide a basis for the mechanism of RYNM treatment of pneumonia.

## Materials and methods

### Database and software application

Using the TCM Database System Pharmacology (TCMSP) (http://lsp.nwu.edu.cn/tcmsp.php) combined with literature screening, the effective components and related targets of *T. mongolicum*, *P. cuspidatum*, *S. oleraceus* and *S. barbata* were searched using a filter condition for OB (oral drug bioavailability) >30%; DL (class medicine) >0.18 (Li et al. 2019).

Information on potential targets for pneumonia is available at DrugBank (https://www.drugbank.ca/), DisGeNET (http://www.disgenet.org/web/DisGeNET/menu) and CTD (https://ctdbase.org/). From the information obtained from the database, the obtained target name was input into the UniProt database to obtain the gene name of the target (Gene name) (Song et al. 2018). A Venn diagram was used to map the intersection of drug-related targets and potential targets of the disease to obtain active targets that may have an effect.

The ClusterProfiler package in RStudio software was used for KEGG metabolic pathway analysis and GO enrichment analysis. The active target of drug action was input into the STRING database to construct protein interaction network (PPI) analysis. The network of ‘active component-active target-metabolic pathway’ of RYNM in the treatment of pneumonia was constructed by Cytoscape 3.7.0.

### Drugs and microorganisms

According to the 2015 Chinese Pharmacopoeia, *T. mongolicum*, *P. cuspidatum*, *S. oleraceus* and *S. barbata* medicinal materials were purchased from Xi'an Chanba Chinese herbal medicine market in August 2019. The above medicinal materials were identified by Professor Benxiang Hu of the School of Pharmacy, Shaanxi University of Chinese Medicine, and were considered to be genuine. Each voucher specimen was deposited at the College of Pharmacy of Shaanxi University of Chinese Medicine. The voucher specimen numbers of *T. mongolicum*, *P. cuspidatum*, *S. oleraceus* and *S. barbata* are no. H20190812, no. R20190905, no. H20190827 and no. H20190911, respectively. The herbs in the RYNM prescription are extracted with water according to the proportion, and then concentrated into a relative density of 1.16 g/mL. Levofloxacin hydrochloride tablets, which were provided by Sichuan Kelun Pharmaceutical Co., Ltd. (B181202C32), were dissolved in purified water using an ultrasonic device, resulting in a drug content of 10 mg/mL.

*Streptococcus pneumonia* was purchased from Beijing Biobw Biotechnology Co., Ltd. (bio-00005, Beijing, China) and was inoculated onto a blood agar plate (Beijing Land Bridge Technology Co., Ltd., Beijing, China, 190907) and cultured at 37 °C for 20–24 h. IL-10 (# SU-B30194), NOS2 (# SU-B36839), COX-1 (# SU-B36877), IL-6 (# SU-B30219), TNF-α (# SU-B31063) and NF-κB (# SU-B35496) ELISA kits were purchased from Jianglai Biological (Shanghai, China). PVDF membrane (# IPVH00010) was purchased from Millipore (Billerica, MA). Sheep anti-rabbit IgG-HRP (# WLA023), TNF-α antibody (# WL01581) and IL-6 antibody (# WL02841) were purchased from Shenyang Wanleibio Co., Ltd. (Shenyang, China).

### Experimental animal

SPF-grade male Sprague Dawley (SD) rats weighing 180–220 g were selected and provided by Chengdu Dossy Experimental Animals Co., Ltd. (Chengdu, China). The feeding environment consisted of 18–26 °C, relative humidity of 40–70%, and ventilation 8–12 times/h. Rats had *ad libitum* access to drinking water (pure water) and feed (Chengdu Dossy Experimental Animals Co., Ltd., Chengdu, China).

After 1 week of adaptive feeding, 40 SD rats were selected and randomly divided into four groups of 10 rats: control, model, levofloxacin and RYNM groups. The rats in each group were given an intratracheal injection of 0.1 mL of 1 × 10^8^ CFU/mL *Streptococcus pneumonia* bacterial solution once a day for three days (Borsa et al. 2019). Rats in the levofloxacin group received oral administration of levofloxacin 75 mg/kg, and rats in the RYNM group received oral administration of RYNM 15 g/kg (Lyu et al. 2020). The drug dose conversion formula was as follows: human dose of crude herbs on clinic × 0.018/200 × 1000 × the multiple of clinical equivalent dose. The model group was given normal saline as placebo. Normal saline was also given to the control group by tracheal infusion and oral administration. The rats in each group were given 10% chloral hydrate (3 mL/kg) by peritoneal injection for anaesthesia 24 h after the last dose. Blood (5 mL) was collected from the abdominal aorta and placed in an anticoagulant vacuum blood container without additives. The blood samples were centrifuged at 3000 rpm for 10 min, and the serum was stored at −20 °C for analysis (Guo et al. 2018). The right lung was ligated, and normal saline was injected from the trachea to the left lung twice, 1.5 mL each time. The alveolar rinse solution was collected, centrifuged at 3000 rpm for 10 min, and the supernatant was removed and stored at −20 °C. The upper lobe and middle lobe of the right lung were stored at −80 °C, and the lower lobe and right posterior lobe of the right lung were fixed in 10% formalin solution for subsequent analysis (Qu et al. 2019). The Animal Ethics Committee of Shaanxi University of Chinese Medicine approved all experimental programs involving rats, and all experimental animals and animal experiments were in line with animal experimental procedure ethics.

### Pulmonary histology

Tissues of the lower lobe and posterior lobe of the right lung fixed in 10% formalin solution from each group were dehydrated in an automatic tissue dehydrator with gradient alcohol from low concentration to high concentration, and then embedded with xylene and paraffin; 4 μm tissue wax blocks were cut, dipped in haematoxylin dye and stained for 5 min, then stained with eosin, and the processed sections were histopathologically examined.

### Biochemical analysis

The serum and alveolar lavage fluid samples from each group of rats were re-equilibrated at room temperature for 20 min. The contents of the indicators were determined, the OD value was measured by an enzyme-linked immunoassay instrument, and the corresponding concentration was calculated according to the standard curve.

### Western blot

Tissue samples were mixed with the corresponding volume of lysate, and centrifuged at 12,000 rpm and 4 °C for 10 min, and the supernatant was separated to obtain the protein extract. The SDS-PAGE electrophoresis device was assembled according to the manufacturer’s instructions and the polyacrylamide gel was configured with a concentration of 5%, separation concentration of 10% and 15%, and was poured along one side of the glass plate. The upper layer was sealed with water to promote gel polymerization. When the separation glue was polymerized, the water layer was poured out, and the prepared concentrated glue was poured in. Finally, it was sealed with a comb, and was left for approximately 30 min (Yang et al. 2017). The electrophoresis tank was installed, the electrophoresis solution was injected into the positive and negative electrodes of the electrophoresis tank, 20 μL protein solution was added, a 5 μL protein marker was added, the voltage was adjusted to 80 V, and constant voltage electrophoresis was performed for 2.5 h. The transfer buffer solution was pre-cooled to 4 °C, the transfer filter paper, sponge, etc. were soaked in the transfer buffer solution, a PVDF film of appropriate size for marking was inserted into the transfer tank and the voltage was adjusted to 80 V and transferred for 1.5 h. After the transfer was completed, the PVDF film was removed, immersed in TBST and shaken on a shaker for 5 min. The TBST was decanted and the PVDF membrane was dipped in a skimmed milk powder solution and blocked for 1 h. The antibody was diluted with 5% skimmed milk powder and incubated with primary antibody. The PVDF membrane was washed four times with TBST, the skimmed milk powder diluted with secondary antibody was added, and incubated at 37 °C for 45 min. The membrane was then immersed in TBST, shaken for 5 min and washed six times (Feng et al. 2016). The film was scanned and the optical density value of the target band was analysed by a gel image processing system (Gel-Pro-Analyzer software, Media Cybernetics, Inc., Rockville, MD).

### Statistical methods

Statistical analysis was performed using IBM SPSS 25 (Armonk, NY), and data analysis was performed using the LSD method and T2 (M) in the one-way ANOVA test. *p*< 0.05 was considered statistically significant.

## Results

### Construction of the compound database

**Figure 1.**
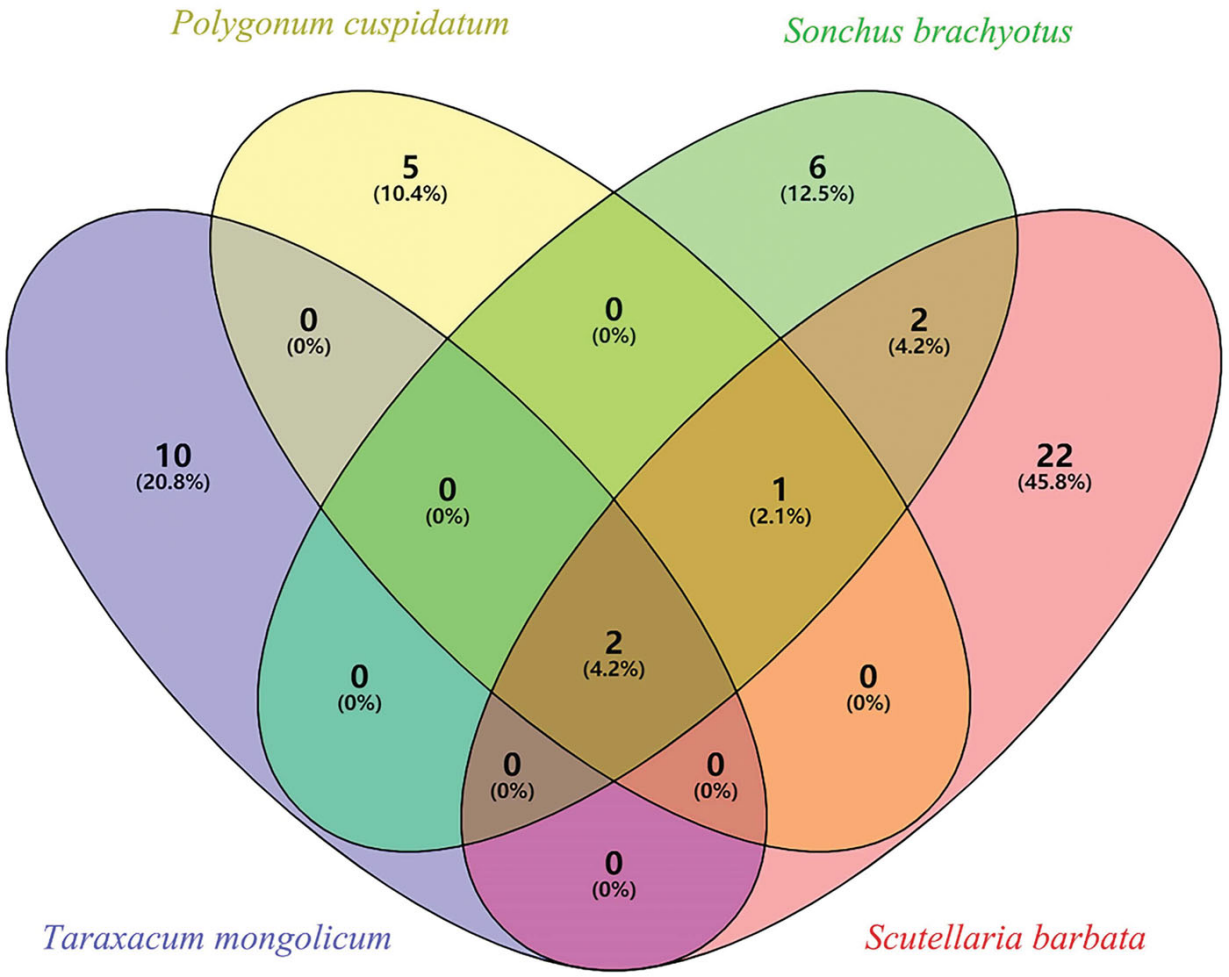
Venn diagram of the components of RYNM.

Using the TCMSP database and literature reports, compounds and targets of *T. mongolicum*, *P. cuspidatum*, *S. oleraceus* and *S. barbata* were screened, and 12 chemical components were found in *T. mongolicum*, eight in *P. cuspidatum*, 11 in *S. oleraceus* and 27 in *S*. *barbata*. A Venn diagram of the medicinal materials is shown in Figure 1. According to the potential targets of pneumonia obtained from the DrugBank, DisGeNET and CTD databases, a total of 48 effective components with therapeutic effect were speculated by mapping with the target of medicinal materials, and the TCMSP number, molecular name and compound classification were obtained, as shown in Table 1.

**Table 1.** Information of active ingredients in RYNM.

Herb name	TCMSP ID	Molecule name	OB (%)	DL
*S. barbata*	MOL001040	(2R)-5,7-Dihydroxy-2-(4-hydroxyphenyl) chroman-4-one	42.36	0.21
	MOL012245	5,7,4′-Trihydroxy-6-methoxyflavanone	36.63	0.27
	MOL012246	5,7,4′-Trihydroxy-8-methoxyflavanone	74.24	0.26
	MOL012248	5-Hydroxy-7,8-dimethoxy-2-(4-methoxyphenyl) chromone	65.82	0.33
	MOL012250	7-Hydroxy-5,8-dimethoxy-2-phenyl-chromone	43.72	0.25
	MOL012251	Chrysin-5-methylether	37.27	0.20
	MOL000173	Wogonin	30.68	0.23
	MOL001735	Dinatin	30.97	0.27
	MOL002714	Baicalein	33.52	0.21
	MOL002915	Salvigenin	49.07	0.33
	MOL000351	Rhamnazin	47.14	0.34
	MOL005190	Eriodictyol	71.79	0.24
	MOL008206	Moslosooflavone	44.09	0.25
	MOL000953	CLR	37.87	0.68
	MOL001973	Sitosteryl acetate	40.39	0.85
	MOL012269	Stigmasta-5,22-dien-3-ol-acetate	46.44	0.86
	MOL012270	Stigmastan-3,5,22-triene	45.03	0.71
	MOL001755	24-Ethylcholest-4-en-3-one	36.08	0.76
	MOL012254	Campesterol	37.58	0.71
	MOL005869	Daucostero_qt	36.91	0.75
	MOL012266	Rivularin	37.94	0.37
	MOL012252	9,19-Cyclolanost-24-en-3-ol	38.69	0.78
*T. mongolicum*	MOL000006	Luteolin	36.16	0.25
	MOL002881	Diosmetin	31.14	0.27
	MOL000008	Apigenin	33.01	0.21
	MOL000415	Rutoside	32.08	0.68
	MOL000098	Quercetin	46.43	0.28
	MOL003837	Esculetin	42.97	0.27
	MOL003319	P-Hydroxyphenylacetic acid	41.89	0.21
	MOL000105	Protocatechuic acid	35.37	0.19
	MOL000414	Caffeic acid	54.97	0.25
	MOL000360	Ferulic acid	39.16	0.19
	MOL003871	Chlorogenic acid	53.61	0.31
	MOL000040	Scopoletol	37.77	0.22
*S. oleraceus*	MOL001689	Acacetin	34.97	0.24
	MOL002322	Isovitexin	31.29	0.72
	MOL000422	Kaempferol	41.88	0.24
	MOL001678	Bolusanthol B	39.94	0.41
	MOL000449	Stigmasterol	43.83	0.76
	MOL000359	Sitosterol	36.91	0.75
	MOL001697	Sinoacutine	63.39	0.53
	MOL001677	Asperglaucide	58.02	0.52
*P. cuspidatum*	MOL000358	β-Sitosterol	36.91	0.75
	MOL000492	(+)-Catechin	54.83	0.24
	MOL013288	Picralinal	58.01	0.75
	MOL013281	6,8-Dihydroxy-7-methoxyxanthone	35.83	0.21
	MOL002268	Rhein	47.07	0.28
	MOL013287	Physovenine	106.21	0.19

Of these components, chlorogenic acid, which is a polyphenolic natural compound, is commonly found in apples, coffee beans, grapes, pulp, peels and tea. It is structurally a depsipeptide produced from caffeic acid and quinic acid. It is reported that it has a number of health benefits, including antibacterial, antiviral, antitumor, increases white blood cells, blood pressure activity, blood lipid activity, scavenges free radicals, central nervous system excitation and other biological activities (Hua et al. 2008). Chlorogenic acid has bacteriostatic effects on Gram positive bacteria (*Staphylococcus aureus*, *Bacillus subtilis*) and Gram negative bacteria (*Shigella, Escherichia coli*, *Salmonella typhi*). It has the strongest inhibitory effect on *Streptococcus pneumonia* and *Shigella* (Lou et al. 2011). Chlorogenic acid may induce the immediate and massive release of K^+^ from *Shigella* and *Streptococcus pneumonia* by increasing the permeability of the plasma membrane, which may cause significant nucleotide leakage (Yang et al. 2012).

Many biological studies have shown that apigenin has significant anti-inflammatory, antioxidant and anticancer properties, and can regulate cell proliferation, cell invasion, angiogenesis and apoptosis, by regulating NO, TNF-α, IL-1β and IL-6 related mRNAs in a dose-dependent manner (Song et al. 2016; Chen et al. 2020). Of these factors, NO plays a particularly important role in the immune response to inflammation, and other factors are important for the prevention and treatment of tumours (Balez et al. 2016; Luan et al. 2016). It has been reported that apigenin can restore the increase in apoptosis-related factors such as Cyt-c, Bax, Caspase-9 and Caspase-3 induced by acrylonitrile to some extent (Zhao et al. 2019).

Rhein can effectively block the ATP-induced increase in [Ca^2+^] in a dose-dependent manner (Hu et al. 2019). In addition, rhubarb inhibits the production of ATP-induced intracellular reactive oxygen species (ROS) induced by P2X_4_-mediated Ca^2+^ entry into synovial cells (Khan et al. 2019). Moreover, in lipopolysaccharide (LPS)-treated cells, the application of ATP synergistically promoted gene expression of COX-2, IL-6 and MMP-9 (Yang et al. 2019). Rhein attenuates the expression of these inflammatory genes and increases their expression through ATP.

### Construction of the medicinal material-component-target protein network

**Figure 2.**
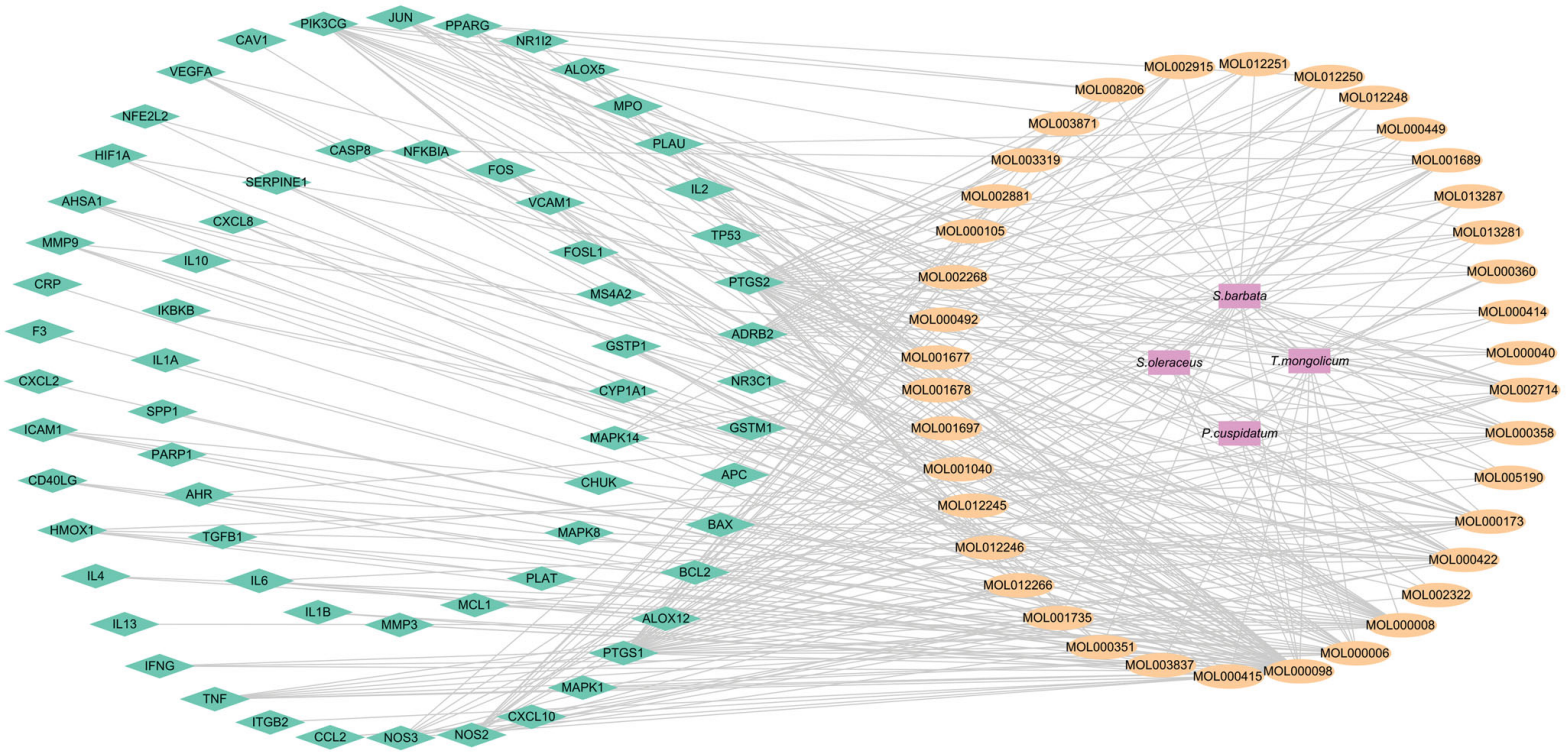
Network diagram of ‘medicinal materials-ingredients-targets’ of RYNM. The squares represent the medicinal materials in RYNM, the ovals represent the TCMSP chemical composition numbers, and the diamonds represent the targets.

Cytoscape 3.7.0 software was used to map the network of ‘Chinese herbs – active components – potential targets of RYNM in the treatment of pneumonia (Figure 2). According to the results, the compatibility of TCM indicated that there may be a synergistic effect between the effective ingredients and multiple targets. For example, chlorogenic acid, ferulic acid and protocatechuic acid in *T. mongolicum* have a regulatory effect on the active target PTGS2; Rhein and (+)-catechin in *P. cuspidatum* are involved in the regulation of PTGS1, HSP90 and NCOA2 target proteins; the active ingredients kaempferol, β-sitosterol and stigmasterol in *S. oleraceus* have a regulatory effect on targets such as ADRB2, PTGS2, PLAU, NOS3 and BAX; in *S. barbata*, rivularin and baicalein regulate NOS2, VEGFA and BCL2. It was indicated that the combination had a stronger effect than that of single drug. Quercetin and luteolin are common components in the four herbs, which regulate PTGS1, HSP90, MAPK1, IL-10, TNF, JUN, IL-6 and other targets, suggesting that the combined use of these four herbs increases the content of their effective components, which is consistent with the ‘phase need’ in the compatibility theory of TCM, and can enhance their therapeutic effect.

### Protein–protein interaction network analysis

**Figure 3.**
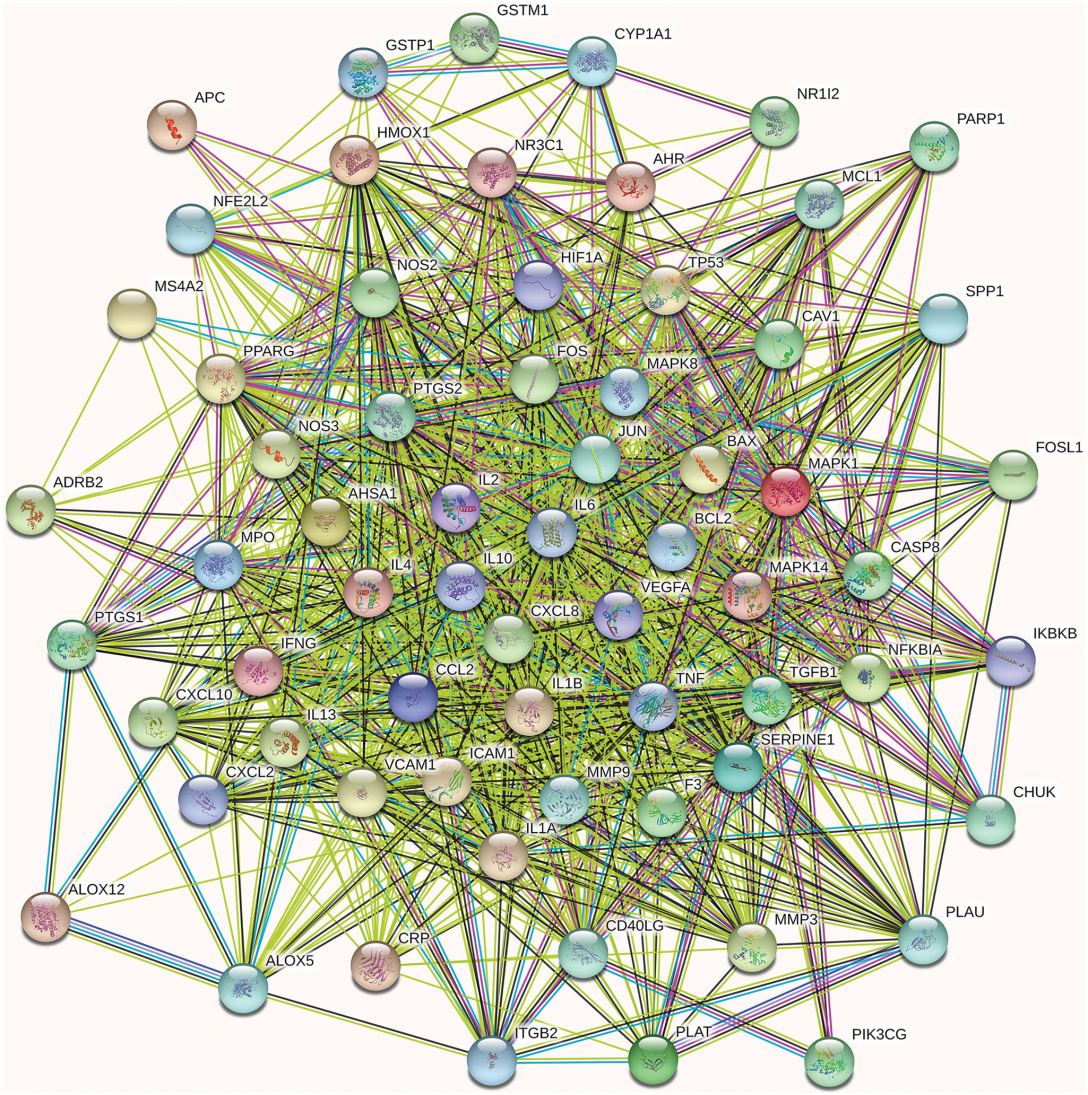
PPI network diagram of RYNM active targets.

The target of RYNM and the potential target of pneumonia were mapped with Venn 2.1 to obtain a total of 65 targets. The target intersection was entered into the STRING database, and the species was set to ‘*Homo sapiens*’ for PPI analysis (Figure 3).

There are a total of 895 edges and 60 nodes in this network. We found that IL-6, PTGS2, TNF, JUN, IL-1B and other target proteins had higher scores and were more connected to other targets. According to research reports, IL-6 has a variety of biological functions, acting on B cells, T cells, liver cells, haematopoietic cells and central nervous system cells. It is a powerful inducer of acute reactions and induces differentiation in B cells. It plays an important role in Ig secreting cells and is involved in the differentiation of lymphocytes and monocytes (Arkatkar et al. 2017). PTGS2 is responsible for the production of inflammatory prostaglandins, and the upregulation of PTGS2 is also related to increased cell adhesion, phenotypic changes, anti-apoptosis and tumour angiogenesis (Astakhova et al. 2019).

### KEGG pathway and GO enrichment analysis

**Figure 4.**
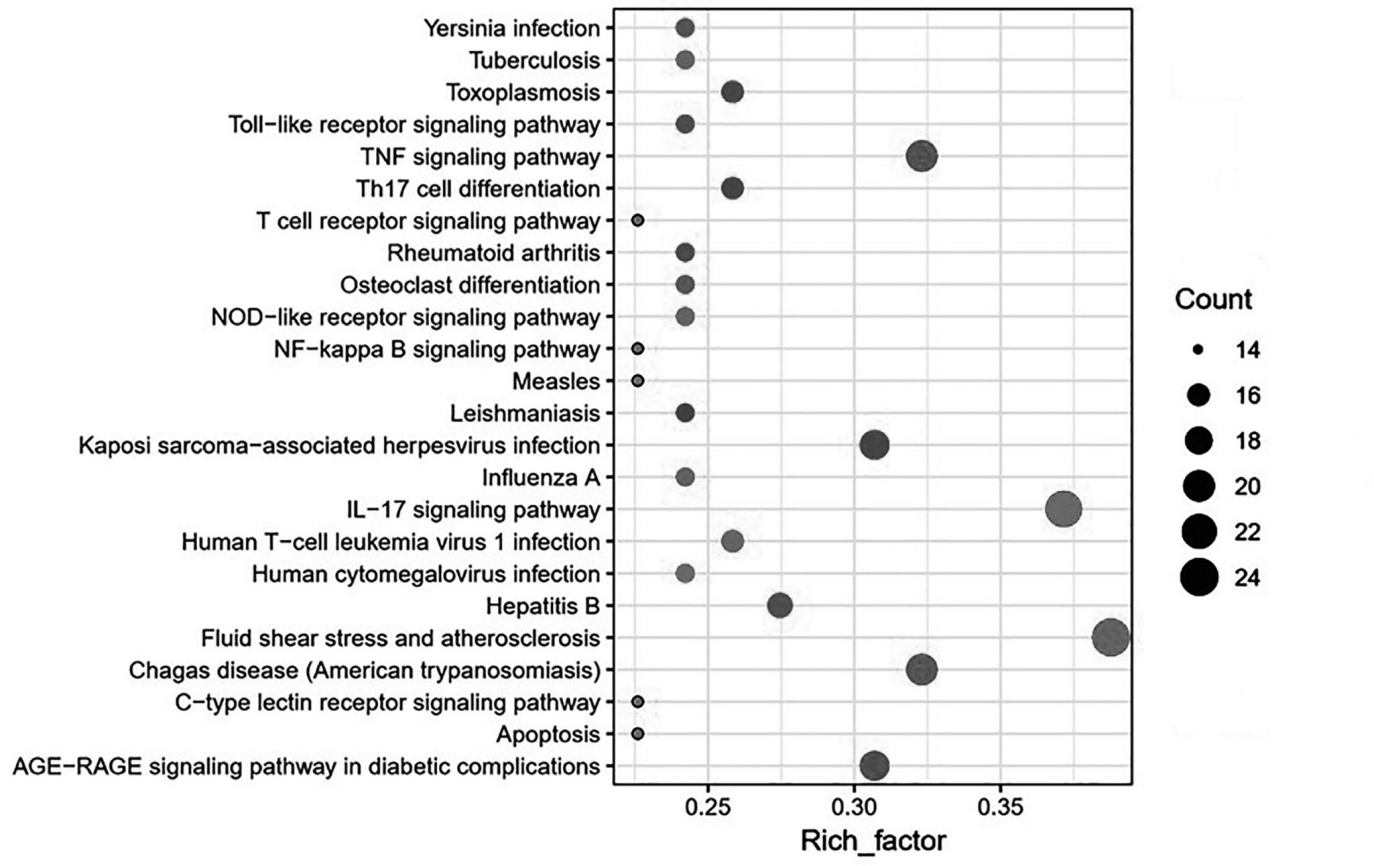
Bubble analysis of the active target KEGG metabolic pathway. The size of the dot represents the number of target proteins enriched in the pathway.

Bio-informatics analysis is based on the R language Bioconductor software package, statistical analysis and visualization of functional clustering of gene sets or gene clusters. The gene name is converted to ‘ENTREZID’, Org Db is selected as ‘org.Hs.eg.db’; the *p* value Cutoff = 0.05 for KEGG pathway enrichment analysis, and the top 20 pathways are filtered to draw a bubble map (Figure 4).

According to the analysis results, there are 23 targets involved in the IL-17 signalling pathway, and it has a smaller *p* value, which fully shows that it plays an important role in both acute and chronic inflammatory reactions. The IL-17 family is a subset of cytokines composed of IL-17A-F, a hallmark of the newly defined T helper 17 (TH17) cell subset, plays an important role in protecting the host against extracellular pathogens, and promotes inflammatory pathology in autoimmune diseases, while IL-17F is mainly involved in mucosal host defence mechanisms. The IL-17 family signals through its corresponding receptors and activates downstream pathways including NF-κB, MAPK and C/EBP to induce the expression of antimicrobial peptides, cytokines and chemokines (Wu et al. 2015). The receptor proximal adaptor Act1 (an NF-κB activator 1) is considered the master mediator in IL-17A signalling.

There are 24 targets involved in the fluid shear stress and atherosclerosis pathway. Fluid shear stress represents the friction force exerted by blood flow on the endothelial surface of the vascular wall. It plays a core role in vascular biology and can up-regulate the expression of endothelial cell (EC) genes and proteins (Lee et al. 2018). The activation of NF-κB and activator protein 1 (AP-1) can reflect the disordered state of ECs. These genes and proteins can prevent atherosclerosis and promote the oxidation and inflammation of the arterial wall (Chatzizisis et al. 2007).

There are 20 targets involved in the TNF signalling pathway. As an important cytokine, TNF can induce a variety of intracellular signalling pathways, including apoptosis and cell survival, inflammation and immunity (Shuh et al. 2013). Activated TNF assembles into homotypic trimers and binds to its receptors TNFR1 and TNFR2, leading to trimerization of TNFR1 or TNFR2. TNFR1 is expressed in almost all cells and is the main receptor for TNF-α. TNFR2 is a receptor for TNF-β and is expressed in a limited number of cells, such as CD4 and CD8 T lymphocytes, ECs, microglia, neuron subtypes, cardiomyocytes, thymocytes and human mesenchymal stem cells. After binding to the ligand, TNFR1 signalling induces the activation of many genes that are mainly controlled by two different pathways, namely the NF-κB pathway and the cascade of MAPK or apoptosis and necrotic necrosis (Lv et al. 2017). TNFR2 signalling activates the NF-κB pathway, including the PI3K-dependent NF-κB pathway and JNK pathway leading to survival.

Of the top 24 pathways screened, 17 were associated with inflammation, regulating immune and pro-inflammatory cytokines, respectively, of which NF-κB has a dimeric effect on immune function, inflammation and cell survival. Activation of NF-κB by TNF-α, IL-1 or by-products of bacterial and viral infection is an exemplary pathway. *In vivo*, Th17 differentiation requires antigen presentation and co-stimulation, and activation of antigen-presenting cells (APCs) to produce TGF-β, IL-6, IL-1, IL-23 and IL-21 (Meka et al. 2015), and has an anti-inflammatory function.

**Figure 5.**
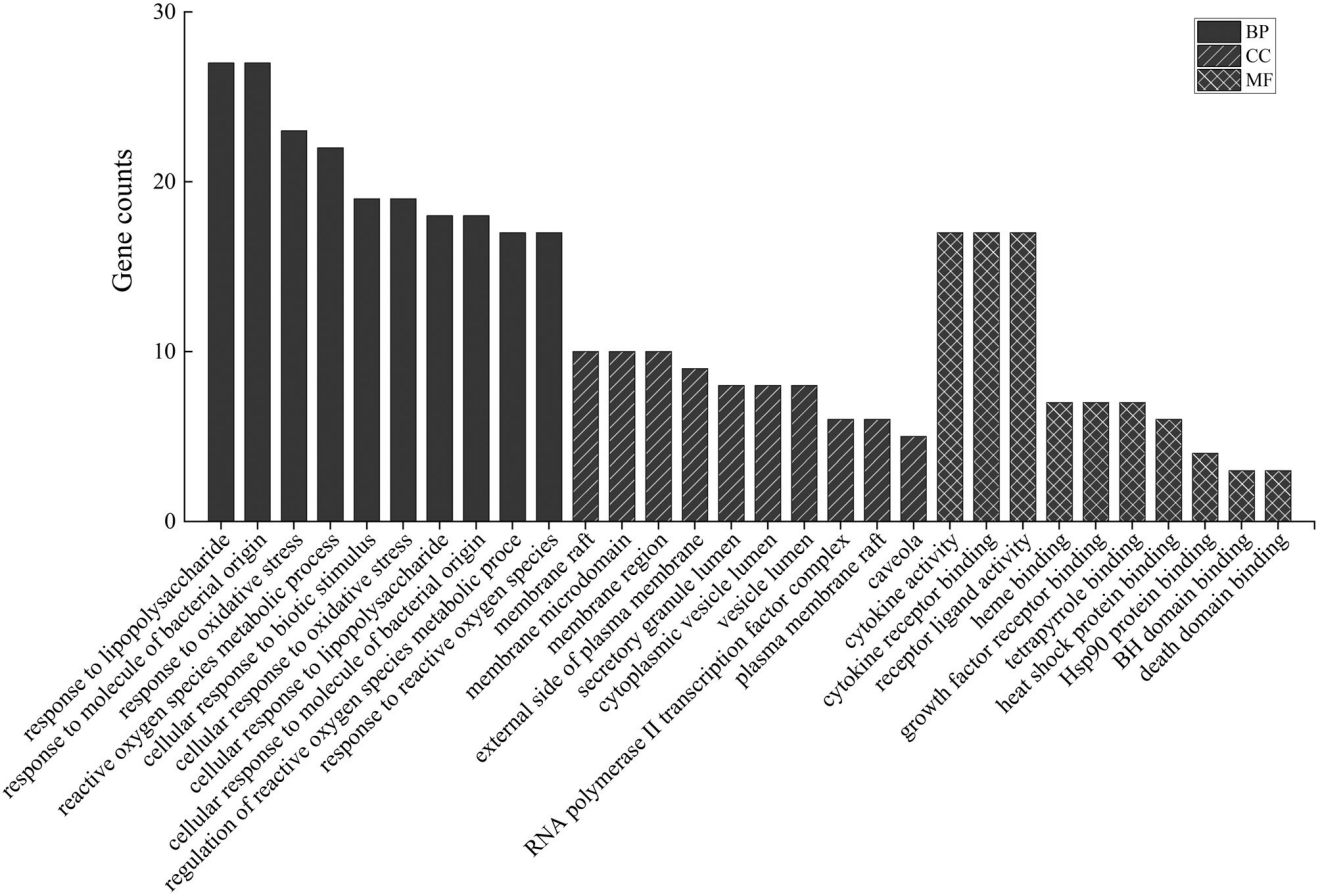
Bar graph for GO enrichment analysis of active targets.

Gene Ontology describes the biological processes (BPs) involved in proteins or RNA, the location of the cells, information on the molecular functions (MFs), and the functional concepts of different conceptions into a directed acyclic graph (DAG) structure (Tian et al. 2019). GO enrichment analysis of potential target proteins for pneumonia was performed by ClusterProfiler, and a *p* value Cutoff = 0.05 was set for BPs, cell components (CCs) and MF analysis; *Q* value Cutoff = 0.01 and a bar graph was drawn (Figure 5). GO enrichment analysis yielded a total of 1110 entries. The enrichment analysis results were mainly focussed on BPs, with a total of 1000 enrichment results. Among them, positive regulation of protein oligomerization is a process that activates or increases the frequency, rate or degree of oligomerization of a protein. Regulation of heterotypic cell–cell adhesion is a process that regulates the frequency, rate or extent of heterotypic cell-to-cell adhesion. It also includes processes such as positive regulation of haemostasis, the organic acid biosynthetic process and the carboxylic acid biosynthetic process. Twenty-six results were enriched in CCs, including membrane raft, membrane microdomain and plasma membrane raft. There were 84 enrichment results in MF, which mainly involved functions such as cytokine activity, cytokine receptor binding and receptor ligand activity. The results suggested that the key targets related to RYNM for pneumonia may be related to cytokine activity, cytokine receptor binding, plasma membrane, cell nucleus and other biological functions.

### Construction of the ‘active ingredient – potential target – metabolic pathway’ network

**Figure 6.**
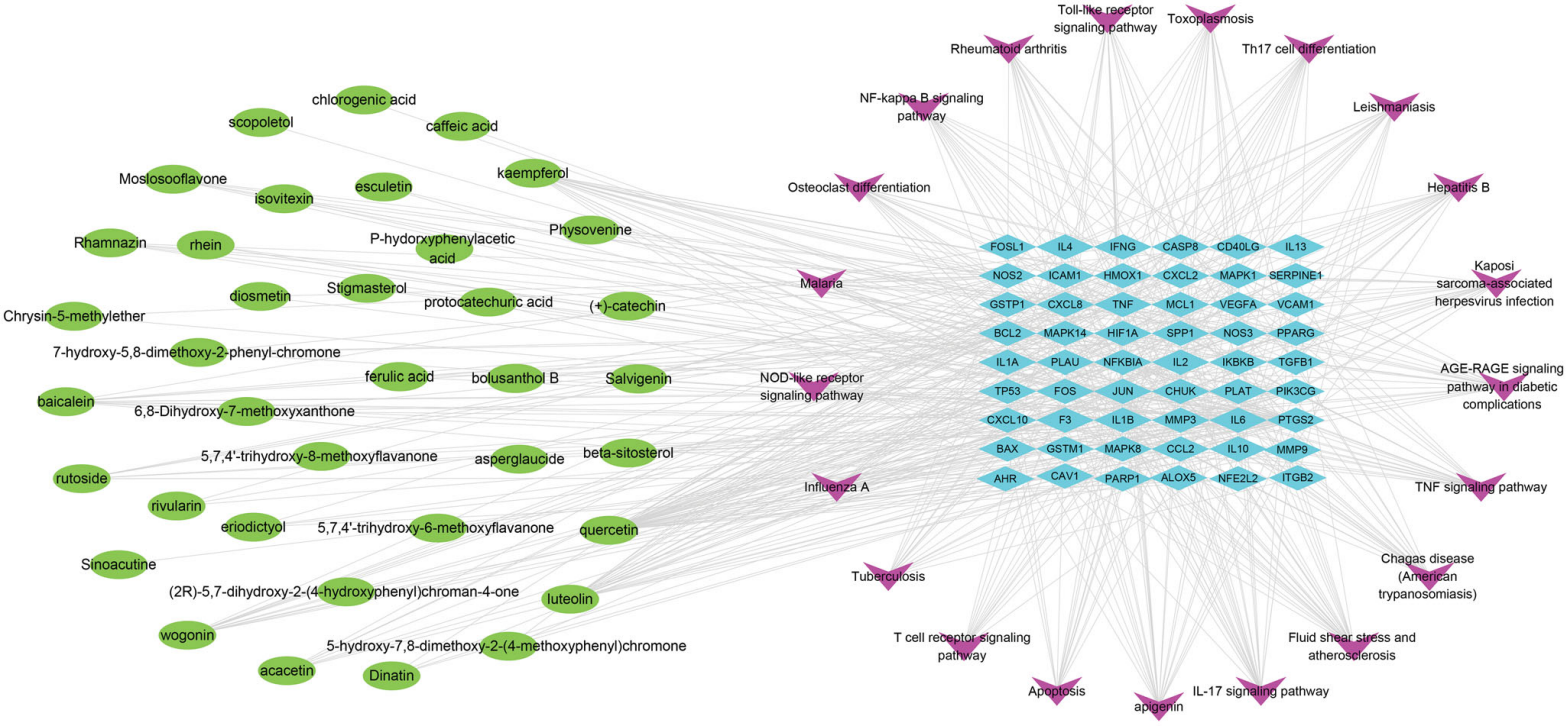
‘Composition-target-path’ network diagram of RYNM. The ovals represent the chemical constituents in RYNM, the diamonds represent potential targets and the inverted triangles represent metabolic pathways.

Based on the results of the target protein enrichment in the Bioconductor package, the top 20 metabolic pathways were screened according to the number of gene entries in the pathway, and the chemical components of the target protein enriched in each pathway were mapped to assess their involvement in regulation compounds of proteins constructed according to the relationship between the ‘chemical composition-target protein-metabolic pathway network diagram’ (Figure 6). The results showed that 38 compounds in RYNM are involved in the regulation of related target proteins and regulate the IL-17 signalling pathway, TNF signalling pathway, NF-κB signalling pathway and other related pathways to play a role in treating pneumonia. Among them, 23 target proteins such as PTGS2, TNF and FOS were enriched in the IL-17 signalling pathway, indicating that the IL-17 signalling pathway plays an important role in the treatment of pneumonia by RYNM. In addition, kaempferol, quercetin, rutoside and other compounds regulate the fluid shear stress and atherosclerosis pathway by acting on IL-1B, JUN and BCL2 target proteins, indicating that the combined use of drugs has a synergistic effect, which is consistent with the multi-component, multi-target and multidimensional pharmacological effects of TCM in the treatment of diseases.

### Results of lung histopathology

**Figure 7.**
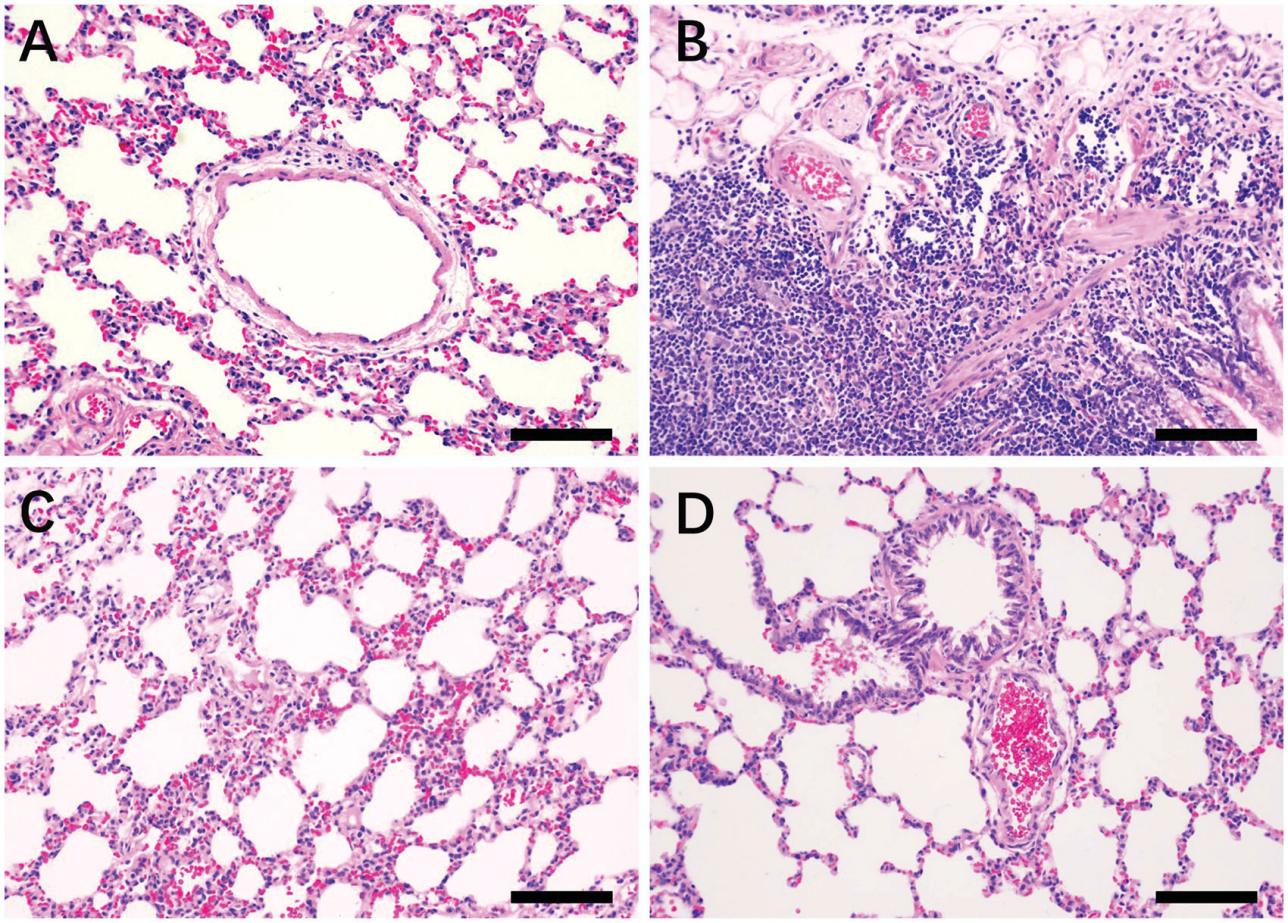
Pathological observation of HE staining in the lung tissue of each group of rats (×200). (A) Control group; (B) model group; (C) levofloxacin hydrochloride group; (D) RYNM group. Severity of lesions in each group: B » C > A>D; infiltration of inflammatory cells: B » C > A>D.

The results of rat lung tissue examination under the microscope are shown in Figure 7. The control group had relatively complete tissue structure, clear staining, uniform cell size, no metastaining and no inflammatory cell infiltration, but there was a slight thickening of the lung interstitium. Histopathologic changes in the model group were obvious, with a few complete alveolar structures, an uneven size of alveolar cells, a different staining pattern, shrunken nuclei, a pulmonary interstitial area with obvious hyperplasia and fibrosis, accompanied by small blood vessels, inflammatory cell hyperplasia, mainly neutrophils and lymphocytes and few macrophages. The tissue structure in the levofloxacin group showed slight lesions, with varying degrees of lung interstitium thickening, alveoli of different sizes, and other aspects were not obvious. Only scattered individual inflammatory cells existed. The RYNM group showed normal lung tissue structure, no obvious pathological changes in the alveoli and trachea, clear cell staining, uniform cell size, slightly thickened interstitium in some areas, and no inflammatory infiltration in the tissue.

### Expression of inflammatory factors in rat serum and alveolar lavage fluid

**Figure 8.**
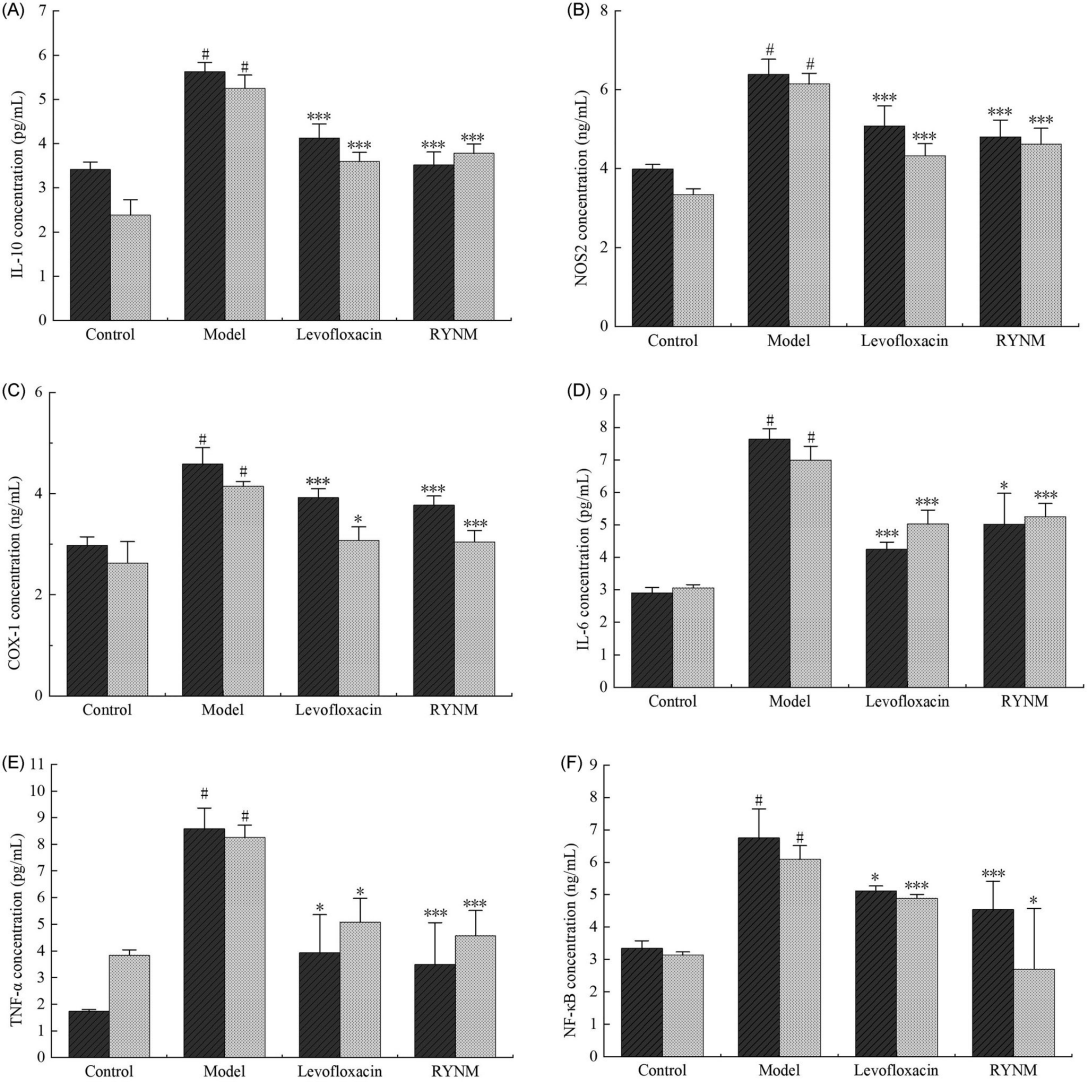
ELISA results of rat serum and alveolar lavage fluid. The black bars represent the expression levels of factors in serum, and the gray bars represent factor levels in alveolar lavage fluid. Data were expressed as the mean ± S.E.M. (*n* = 10). ^#^*p* < 0.05 vs. control group. *p < 0.05, ***p < 0.001 vs. model group.

The expression levels of IL-10, NOS2, COX-1, IL-6, TNF-α and NF-κB in rat serum and alveolar lavage fluid were determined based on the results of network pharmacological prediction (Table 2). ANOVA results showed that compared with the normal control group, the levels of IL-10, NOS2, COX-1, IL-6, TNF-α and NF-κB in serum and alveolar lavage fluid were significantly increased. There were significant differences in the model group (*p*< 0.05; Figure 8). Compared with the RYNM group, the content of inflammatory factors in the model group decreased significantly, and the contents of IL-10, NOS2, COX-1, TNF-α and NF-κB were significantly different (*p*< 0.01). Compared with the model group, the contents of inflammatory factors in the RYNM group were significantly reduced, and the contents of IL-10, NOS2, COX-1, TNF-α and NF-κB were significantly different (*p*< 0.01). In serum, the contents of inflammatory factors in the RYNM group and the levofloxacin group decreased significantly. The TNF-α concentration in the RYNM group was 3.49 ± 1.56 pg/mL, significantly lower than that in the model group (8.58 ± 0.78 pg/mL), the COX-1 concentration was 3.77 ± 0.18 ng/mL, significantly lower than that in the model group (4.58 ± 0.33 ng/mL), and the IL-10 concentration was 3.52 ± 0.29 pg/mL. COX-1, IL-6 and NF-κB were statistically significant, and IL-6 was significantly different to that in the model group (*p*< 0.05). In serum, the content of inflammatory factors in the model group and the levofloxacin group was significantly reduced. The study showed that levofloxacin had a strong antibacterial effect, and has been shown to have strong antibacterial activity against Gram positive bacteria such as *Staphylococcus aureus* and *Streptococcus pneumonia* (Zusso et al. 2019). A large number of inflammatory factors were also detected in rat alveolar lavage fluid in the model group compared with the RYNM group, and the content of IL-10, NOS2, COX-1, IL-6 and TNF-α was significantly decreased (*p*< 0.01). When the RYNM group and levofloxacin group were compared, the differences in contents were small, RYNM can promptly lower the expression of inflammatory factor levels and may have a significant curative effect in the treatment of pneumonia.

**Table 2.** Effect of RYNM on serum IL-10, NOS2, COX-1, IL-6, TNF-α and NF-κB levels in pneumonia model rats (mean ± S.D., *n* = 10).

		IL-10 (pg/mL)	IL-6 (pg/mL)	TNF-α (pg/mL)
Control	Serum	3.42 ± 0.16	2.91 ± 0.17	1.73 ± 0.07
	BALF	2.38 ± 0.35	3.06 ± 0.10	3.83 ± 0.21
Model	Serum	5.63 ± 0.21	7.64 ± 0.32	8.58 ± 0.78
	BALF	5.25 ± 0.30	6.99 ± 0.43	8.25 ± 0.46
Levofloxacin	Serum	4.13 ± 0.32	4.25 ± 0.22	3.93 ± 1.43
	BALF	3.60 ± 0.21	5.03 ± 0.43	5.08 ± 0.89
RYNM	Serum	3.52 ± 0.29	5.02 ± 0.95	3.49 ± 1.56
	BALF	3.78 ± 0.21	5.25 ± 0.40	4.57 ± 0.94
		NF-κB (ng/mL)	NOS2 (ng/mL)	COX-1 (ng/mL)
Control	Serum	3.34 ± 0.23	3.99 ± 0.12	2.98 ± 0.17
	BALF	3.14 ± 0.10	3.34 ± 0.15	2.63 ± 0.42
Model	Serum	6.75 ± 0.90	6.39 ± 0.38	4.58 ± 0.33
	BALF	6.09 ± 0.42	6.14 ± 0.27	4.14 ± 0.10
Levofloxacin	Serum	5.12 ± 0.15	5.08 ± 0.50	3.92 ± 0.18
	BALF	4.89 ± 0.12	4.32 ± 0.31	3.07 ± 0.27
RYNM	Serum	4.54 ± 0.88	4.80 ± 0.43	3.77 ± 0.18
	BALF	2.70 ± 1.88	4.62 ± 0.40	3.05 ± 0.22

### Protein expression in rat lung tissue by Western blotting

**Figure 9.**
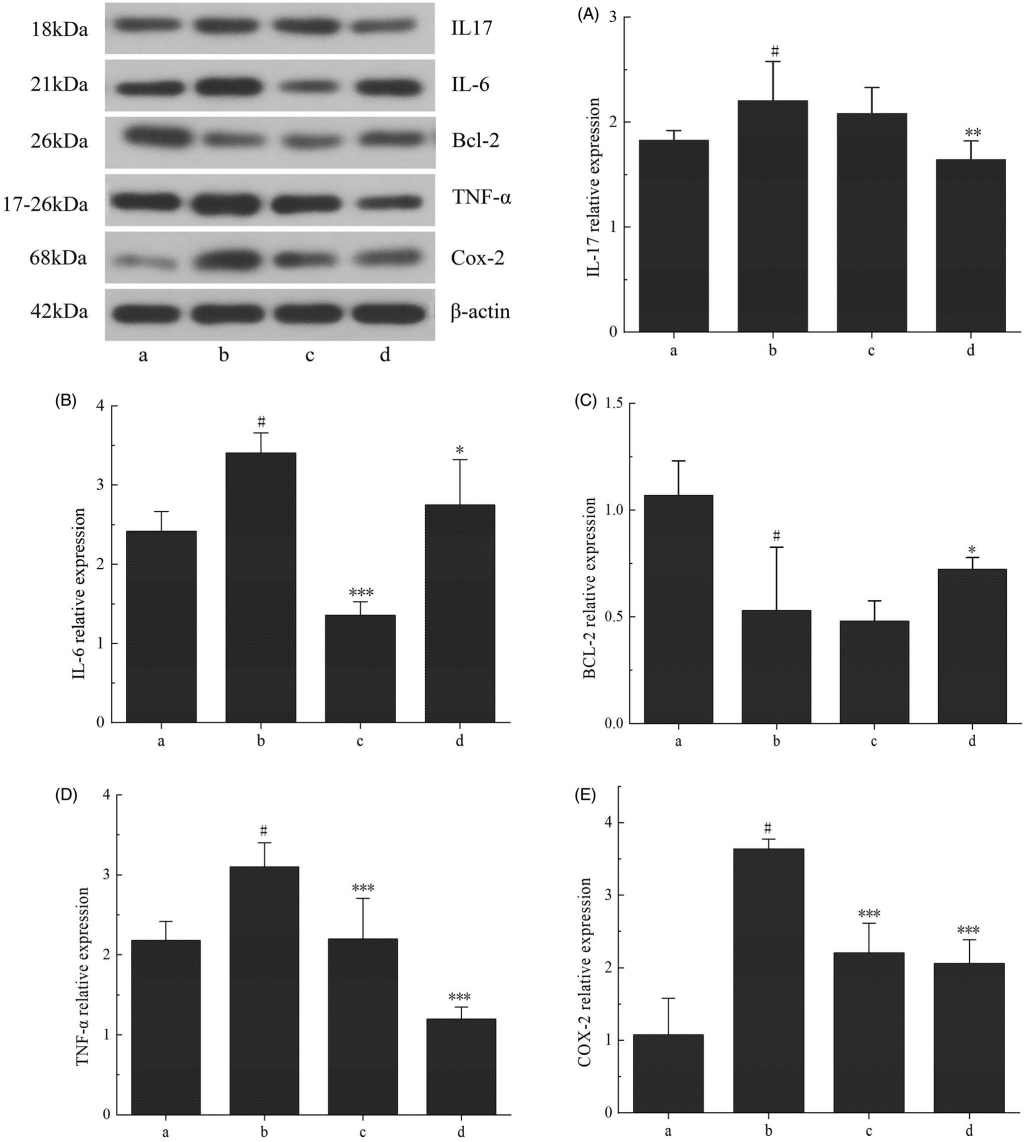
Western blot of rat lung tissue. (a) The control group, (b) the model group, (c) the levofloxacin group and (d) the RYNM group. Data were expressed as the mean ± S.E.M. (*n* = 10). ^#^*p* < 0.05 vs. control group. *p < 0.05, **p < 0.01, ***p < 0.001 vs. model group.

Western blot was performed to determine the expression of inflammatory factors in rat lung tissues to verify the network pharmacological prediction results. The expression of Bcl-2 in the control group was significantly different compared with the model group (*p*< 0.05) (Figure 9). The expression levels of proinflammatory cytokines IL-17, IL-6, TNF-α and COX-2 in lung tissues were significantly lower than those in the model group (*p*< 0.001), and the expression of the anti-apoptotic gene Bcl-2 was slightly higher than that in the model group (*p*< 0.05). Studies have found that Bcl-2 can inhibit cell death caused by a variety of cytotoxic factors, and overexpression of Bcl-2 can enhance the resistance of cells to most cytotoxic factors (Hardwick and Soane 2013). There was no significant difference in IL-17 expression between the levofloxacin group and the model group, but the expression level in the RYNM group was significantly decreased. IL-17 is an early promoter of T-cell-induced inflammatory response, which can be amplified by promoting the release of pre-inflammatory cytokines. When IL-17 binds to the receptor, it can exert its biological role through the MAP kinase pathway and the NF-κB pathway, and can effectively mediate the neutrophil excitatory process, thereby mediating the tissue's inflammatory response (Boonpiyathad et al. 2019). The expression levels of TNF-α and COX-2 were significantly different to those in the model group. During inflammation, the expression levels of TNF-α and COX-2 in inflammatory cells increase, leading to inflammatory reactions and tissue damage. Overall, RYNM had a strong anti-inflammatory effect and can play a role in treating pneumonia by regulating the expression of inflammatory and pro-apoptotic factors.

## Discussion

At present, pneumonia is mainly treated with penicillin and cephalosporin antibiotics. However, the abuse of antibiotics has led to many safety problems, as they can easily cause liver and kidney damage, gastrointestinal tract symptoms and allergic reactions. Moreover, large doses for long periods can cause drug resistance, resulting in some refractory infections (Jehan et al. 2019). TCM is based on the human body's dialectical treatment and the combination of multiple flavours of herbs to treat diseases. It has a long history of clinical application. RYNM is a national new drug approved by the Chinese Food and Drug Administration. Encouraging results have been achieved in the treatment of simple pneumonia, acute pharyngitis and other diseases. Its pharmacological effects are more complex, with characteristics of multiple components, multiple targets and multiple pathways.

In this study, the OB value and DL value were screened in the TCMSP database to ensure that the chemical components could play their roles *in vivo*, and chlorogenic acid, rhubarb acid, luteolin and other chemical components that might have therapeutic effects in RYNM were obtained. According to reports, luteolin inhibits macrophage phosphorylation, inhibits the activity of the transcription factor NF-κB, and can inhibit the production of cytokines IL-6 and TNF-α by macrophages induced by LPS, increasing IFN-γ, reducing specific Ig-E and infiltration of eosinophils (Jiang et al. 2018). The network of target sites of medicinal ingredients suggested that it could regulate multiple targets such as PTGS1, HSP90, MAPK1, IL-10, TNF, JUN and IL-6, which was consistent with previous studies. Ferulic acid can act on targets such as PTGS1, PTGS2 and NOS3, and has the effects of anti-platelet aggregation, inhibition of platelet serotonin release, enhancement of prostaglandin activity, analgesia and relief of vasospasm (Mahmoud et al. 2020).

Enrichment of target proteins by KEGG and GO demonstrated that target proteins are involved in body-related pathways, among which the IL-17 signalling pathway, fluid shear stress and atherosclerosis signalling pathway, and TNF signalling pathway enriched many targets. In GO analysis, potential targets were found to be related to the regulation of intercellular adhesion rate, positive regulation of angiogenesis, organic acid synthesis, etc., and related to cytokine activity, receptor ligand activity, plasma membrane, cell nucleus and other biological functions. The pathway has a greater correlation with inflammation, suggesting that the chemical components in RYNM can act on related target proteins to exert their anti-inflammatory activities.

Common causes of pneumonia are *Streptococcus pneumonia*, *Klebsiella coli*, *Pseudomonas aeruginosa* and *Bacillus influenzae* (Arancibia et al. 2000). In the confirmatory experiments using tracheal instillation of *Streptococcus pneumonia*, rat models were successfully constructed and HE staining showed that the pneumonia model group showed inflammatory cell infiltration and fibrosis compared with the control group. In the RYNM group, there were no obvious pathological changes in the alveoli and trachea, and the degree of inflammatory cell infiltration was significantly reduced compared with the model group. Thus, RYNM has a certain therapeutic effect on pneumonia. ELISA tests of alveolar lavage fluid and serum showed that RYNM significantly reduced the contents of IL-10, NOS2, COX-1, TNF-α and NF-κB, which was consistent with the active targets of network pharmacology. RYNM inhibited the expression of IL-17, IL-6, TNF-α and COX-2 in rat lung tissues, and these targets can reflect inflammation in the body. Cytokines are involved in the entire process of the occurrence and development of pneumonia, and in the innate immune response and adaptive immune response. Bax and Bcl-2 are closely related to cell apoptosis regulation, RYNM can regulate the expression of Bcl-2/Bax, affect respiratory vascular ECs and smooth muscle, has a protective effect on ECs, promotes new cells and the repair process, and reduces local oxidative stress response by regulating target proteins and exerting anti-inflammatory, antibacterial and tissue repair activity (Li et al. 2018).

Network pharmacology provides a new way of thinking about Chinese medical research. Its use for large complex network pharmacology function relationship integration can simplify the difficulty of study, but the comprehensive nature of the TCM database and data reliability are existing problems, as there is a need to filter data in strict accordance with the conditions. Based on the analysis results and *in vivo* validation experiments, the potential active ingredients and mechanism of action of RYNM in the treatment of pneumonia were clarified, providing ideas and methods for studying the mechanism of action of TCM compounds in the treatment of diseases.

Unfortunately, our research also has some limitations. We are unable to provide the HPLC chemical fingerprint of each plant ingredient and RYNM. In the future, the chemical components of Chinese medicine prescriptions will be characterized (HPLC, GC, MS) in order to explore the specific pharmacodynamic material basis of RYNM. We will conduct cell experiments and antibacterial experiments in the future to further explore its potential mechanisms.
